# The 2M6 antigen is a Müller cell-specific intracellular membrane-associated protein of the sarcolemmal-membrane-associated protein family and is also Top_AP_

**Published:** 2010-05-30

**Authors:** Judith D. Ochrietor, Tatiana P. Moroz, Paul J. Linser

**Affiliations:** 1 University of North Florida, Department of Biology, Jacksonville, FL; 2 University of Florida Whitney Laboratory for Marine Biosciences, St. Augustine, FL

## Abstract

**Purpose:**

The differentiation marker 2M6 has been used to identify Müller cells within the developing chick retina for several years, although the molecular identity of 2M6 was not known. This study was aimed at determining the identity of the protein antigen recognized by the 2M6 monoclonal antibody.

**Methods:**

Affinity chromatography and subsequent mass spectrometry were used to determine the molecular identity of the 2M6 antigen. Immunohistochemistry of monolayer preparations and paraffin-embedded sections of chick retina were performed to localize expression of the 2M6 antigen within cells of the chick retina.

**Results:**

Mass spectrometry analyses revealed that the 2M6 antigen is identical (with 95% probability) to the protein known as Top_AP_, which is a member of the sarcolemmal membrane-associated protein family of proteins. The 2M6 polypeptide is expressed by Müller glial cells as well as boundary cells within the chick retina. Expression localizes to intracellular membrane structures within those cells.

**Conclusions:**

Members of the sarcolemmal membrane-associated protein family of proteins have been implicated in structural and functional roles related to the cytoskeleton and Ca^+2^ release from internal stores. It is thought that 2M6 plays a similar role in Müller cells of the vertebrate retina.

## Introduction

Development of the vertebrate retina proceeds such that mitotic cells leave the cell cycle and differentiate into the various cell types found within the tissue. All retina cells differentiate from a common progenitor cell population; cones are “born” relatively early in development and rods and Müller glial cells are the last cell types produced (reviewed in [1]). The neurons and glial cells rely on topographic cues and expression of differentiation factors to migrate to the appropriate layers of the retina and also for guidance of projections [1]. Often these differentiation factors are used as markers to identify cells within the developing retina.

One such differentiation marker is the 2M6 antigen, which was first identified by Schlosshauer et al. [2]. The 2M6 antigen is a 40–46 kDa protein expressed after major laminations of chick retinal tissue are established [2]. Linser et al. [3] reported the presence of a pool of mitotically active cells that have glial-like qualities and express the 2M6 antigen. It is thought that 2M6 influences glial differentiation in the neural retina [3] and is considered a definitive marker of Müller glia [3,4].

In 1995 Savitt et al. [5] reported that Top_AP_ is expressed during periods of retinotectal synapse formation in the chick retina. In embryonic day 8 (E8) chick retina, Top_AP_, a 40-kDa protein, has graded expression along the anterior to posterior axis in retina and optic tectum [5]. Indeed, the name refers to the fact that the protein is a topographic marker expressed along the anterior-posterior axis [5]. Hydropathy plot analyses of the translated cDNA sequence of Top_AP_ suggest that it is a membrane-associated protein [5]. Savitt et al. [5] proposed that Top_AP_ is crucial for synapse connectivity within the developing neural retina.

Presently, we report that Top_AP_ is the 2M6 antigen. Affinity purification of detergent-treated chick retina lysates and subsequent mass spectrometry (MS/MS) analysis indicate that the protein recognized by the 2M6 antibody is identical to the protein named Top_AP_. Liquid chromatography (LC) and and tandem MS/MS were performed at the University of Florida biotechnology core facility in Gainesville, FL. The immunohistochemical data presented herein indicate that 2M6 (Top_AP_) is an intracellular protein within Müller glial cells. The 2M6 (Top_AP_) protein belongs to a family of proteins associated with intracellular membranes and implicated in structural roles within the cells in which they are expressed.

## Methods

### Animals

Fertilized chicken eggs were obtained from Charles River Laboratories (North Franklin, CT) and incubated in a forced-draft incubator at 37 °C with saturated humidity at the University of Florida Whitney Laboratory for Marine Biosciences, St. Augustine, FL. The care and use of these animals was in accordance with University of Florida Institutional Animal Care and Use Committee (IACUC) regulations and the Guide for the Care and Use of Laboratory Animals published by the Institute for Laboratory Animal Research [6].

### Protein extraction

Retinas from twenty E13 chicken embryos were isolated and homogenized in 10 volumes of lysis buffer (Tris-buffered saline [TBS], 0.1% Triton X-100, 1:1,000 dilution of protease inhibitor cocktail [product #P-8340; Sigma Chemical Co., St. Louis, MO]) based on the wet weight of the tissue. The tissue was disrupted via sonication followed by shaking incubation for 1 h at room temperature. The lysate was cleared by centrifugation at 10,000 xg for 30 min at 4 °C. The supernatant was collected and stored at 4 °C.

### Affinity purification

The 2M6 antigen was purified using 2M6-specific antibody (University of Florida Hybridoma Core #HL 1225) [2] coupled to CNBr-activated sepharose 4B, following the protocol of the manufacturer (Amersham Biosciences, Piscataway, NJ). Extracted proteins were added to the affinity column matrix and allowed to incubate on a rotating shaker tray overnight at 4 °C. The unbound fraction was removed from the column and the resin was washed six times with lysis buffer. The bound fraction was eluted using 0.2 M glycine, pH 2.5 and neutralized with 1 M Tris, pH 8.0. The eluted fractions with significant absorbances at 280 nm were pooled and concentrated.

### Immunoblotting of proteins

Immunoblotting was performed as described [7]. Eluted proteins were separated on a NuPAGE 4%–12% Bis-Tris gel in 2-(N-morpholino)ethane sulfonic acid buffer (Invitrogen Corporation, Carlsbad, CA) and transferred to a nitrocellulose membrane (Osmonics, Minnetonka, MN). Blots were stained with 0.1% fast green in methanol, acetic acid, and H_2_O (5:1:5 based on volume), destained, documented, and blocked with a 2% solution of nonfat dry milk in TTBS (TBS containing 0.1% Tween-20) for 1 h at room temperature. After incubation in blocking buffer, the blots were incubated in 2M6 hybridoma supernatant (1:10 dilution in TTBS; University of Florida Hybridoma Core) for 1 h at 37 °C. Blots were washed and incubated in alkaline phosphatase (AP)-conjugated secondary antibody (Jackson ImmunoResearch Laboratories, West Grove, PA) at a dilution of 1:500 for 1 h at 37 °C. The blots were then incubated in AP substrate (Bio-Rad, Hercules, CA). Protein expression was documented using a scanner (ScanJet 6100C; Hewlitt Packard, Palo Alto, CA), and the figures were assembled using Microsoft PowerPoint software (Redmond, WA).

### Mass spectrometry

The affinity-purified sample was cut from the sodium dodecyl sulfate polyacrylamide gel and submitted for analysis. The gel samples were washed with digestion buffer (100 mM Tris, 1% reduced Triton X-100, 10% acetonitrile, pH 8.0), and reduced by incubation in digestion buffer containing 4.5 mM dithiothreitol for 30 min at 55 °C. Iodoacetic acid (10 mM) was added to the solution, and the sample was incubated for 30 min at room temperature. The sample was placed into fresh digestion buffer containing trypsin (1:50 ratio of trypsin to protein) and incubated overnight at 37 °C. The protein was extracted from the solution with 0.1% trifluoroacetic acid and 50% acetonitrile (ACN), dried, and resuspended in loading buffer.

The enzymatically digested samples were injected onto a capillary trap (LC Packings PepMap300, Dionex, Sunnyvale, CA) and desalted for 5 min with a flow rate of 10 ml/min of 0.1% v/v acetic acid. The samples were loaded onto an LC Packing® C18 PepMap high-performance liquid chromatography (HPLC) column. The elution gradient of the HPLC column started at 3% solvent A, 97% solvent B and finished at 60% solvent A, 40% solvent B for 60 min for protein identification. Solvent A consisted of 0.1% v/v acetic acid, 3% v/v ACN, and 96.9% v/v H_2_O. Solvent B consisted of 0.1% v/v acetic acid, 96.9% v/v ACN, and 3% v/v H_2_O. LC-MS/MS analysis was performed on a hybrid quadrupole time-of-flight (TOF) mass spectrometer (QSTAR; Applied Biosystems, Framingham, MA). The focusing potential and ion spray voltage was set to 275 V and 2600 V, respectively. The information-dependent acquisition mode of operation was employed in which a survey scan from *m/z* 400–1,200 was acquired, followed by collision-induced dissociation of the three most intense ions. Survey and MS/MS spectra for each information-dependent acquisition cycle were accumulated for 1 and 3 s, respectively.

Tandem mass spectra were extracted by ABI Analyst version 1.1 (Life Technologies Corporation, Carlsbad, CA). All MS/MS samples were analyzed using Mascot version 2.0.01 (Matrix Science, London, UK). Mascot was set up to search the NCBI database, assuming the digestion enzyme trypsin. Mascot was searched with a fragment ion mass tolerance of 0.30 Da and a parent ion tolerance of 0.30 Da. Iodoacetamide derivative of cysteine, deamidation of asparagine and glutamine, oxidation of methionine, were specified in Mascot as variable modifications. Scaffold (version Scaffold-01–06–03; Proteome Software Inc., Portland, OR) was used to validate MS/MS-based peptide and protein identifications. Peptide identifications were accepted if they could be established at greater than 95% probability, as specified by the Protein Prophet algorithm [8]. Protein identifications are accepted if they can be established at greater than 99% probability and contain at least two identified unique peptides. Protein probabilities were assigned by the Protein Prophet algorithm [9].

### Immunohistochemical analysis of protein expression

For paraffin sections, chick retina was isolated, fixed with 4% paraformaldehyde in 0.1 M cacodylate (pH 7.4) for 1 h at 4 °C. The tissue was transferred through two 30 min changes of Carnoy's fluid and brought to room temperature. Tissues were transferred to 100% ethanol, cleared with aniline:methylsalicylate (1:1, vol/vol), and embedded in paraffin [7]. Tissues were sectioned 12 μm thick, mounted on gelatin-coated slides, rehydrated with xylene followed by graded alcohols, and transferred to TBS (0.01 M Tris, 0.15 M NaCl, 1.3 mM CaCl_2_), pH 7.4. The tissues were labeled for indirect immunofluorescent localization of Müller cell- and neuron-specific proteins. Briefly, the tissues were incubated in TTBS, containing 2% normal goat serum, in a humid environment for 1 h at 37 °C. The tissues were then incubated in TTBS containing 2% normal goat serum with mouse monoclonal hybridoma supernatants anti-2M6 (immunoglobulin G [IgG]; 1:10; University of Florida Hybridoma Core) [2] and anti-5A11 (immunoglobulin M [IgM]; 1:10; University of Florida Hybridoma Core) [10] and rabbit antichicken neurofilament protein (1:100; a generous gift from G. Bennett) [11], or TTBS containing 2% normal goat serum with mouse monoclonal hybridoma supernatant anti-2M6 (1:10; University of Florida Hybridoma Core; 1:10) [2] and rabbit anti-calbindin (1:100; Millipore, Billerica, MA) [12], or TTBS containing 2% normal goat serum with mouse monoclonal hybridoma supernatant anti-2M6 (IgG; 1:10) [2] and mouse monoclonal hybridoma supernatant antihuman natural killer cell antigen (HNK; IgM; 1:10; American Type Culture Collection, TIB-200) [13] for 1 h at 37 °C. The sections were washed in TBS, followed by incubation for 1 h in TTBS containing 2% normal goat serum with either fluorescein isothiocyanate (FITC)-conjugated peanut agglutinin (20 μg/ml; Vector Laboratories, Burlingame, CA) combined with tetramethyl rhodamine isothiocyanate (TRITC)-conjugated goat antimouse IgG and Cy5-conjugated goat antirabbit IgG or a combination of FITC-conjugated goat antimouse IgG with TRITC-conjugated goat antimouse IgM and Cy5-conjugated goat antirabbit IgG or with FITC-conjugated goat antimouse IgG and TRITC-conjugated goat antimouse IgM. All secondary antibodies were purchased from Jackson ImmunoResearch Laboratories and used at a dilution of 1:250. 4',6-diamidino-2-phenylindole (DAPI; Molecular Probes, Eugene, OR) was added to the final wash steps [7]. Coverglasses were mounted with TBS/glycerol (1:1) containing *p*-phenylenediamine (Sigma Chemical Company) and viewed with a Leica SP2 or a Leica SP5 confocal microscope (Leica Microsystems, Bannockburn, IL). Images were gathered digitally using the Leica confocal software and assembled for publication using Microsoft PowerPoint software (Redmond, WA).

For monolayer preparations, chick retina was dissociated by incubation in 1 U papain per 1 mg tissue in PBS for 1 h at 37 °C [7] and plated in Lab-Tek (Nunc, Rochester, NY) chamber slides. The cells were incubated overnight in Dulbecco's Modified Eagle Medium (DMEM, Invitrogen Corporation, Carlsbad, CA) containing 10% fetal bovine serum (FBS) and antibiotics at 37 °C in 5% CO_2_ to allow the cells to attach to the substrate. The medium was removed, the cells were washed with TBS and then fixed with 4% paraformaldehyde in 0.1 M cacodylate (pH 7.4). The cells were washed with TBS and then incubated in TTBS, containing 2% normal goat serum, in a humid environment for 1 h at 37 °C. The cells were then incubated in TTBS containing 2% normal goat serum with mouse monoclonal hybridoma supernatant anti-2M6 (IgG; 1:10; University of Florida Hybridoma Core) [2] and mouse monoclonal hybridoma supernatant anti-HNK (IgM; 1:10; American Type Culture Collection) [13] for 1 h at 37 °C. The cells were washed in TBS, followed by incubation for 1 h in TTBS containing 2% normal goat serum with FITC-conjugated goat antimouse IgG and TRITC-conjugated goat antimouse IgM secondary antibodies, as described above. The nucleic acid stain 1,5-bis{[2-(di-methylamino) ethyl]amino}-4, 8-dihydroxyanthracene-9,10-dione (DRAQ-5; 1:1,000; Biostatus, Shepshed, UK) was added to the final wash steps [14]. Coverglasses were mounted with TBS/glycerol (1:1 volume) containing *p*-phenylenediamine (Sigma Chemical Company) and viewed with a Leica Microsystems SP2 confocal microscope. Images were gathered digitally using the Leica Microsystems confocal software and assembled for publication using Microsoft PowerPoint software.

## Results

**Figure 1:**
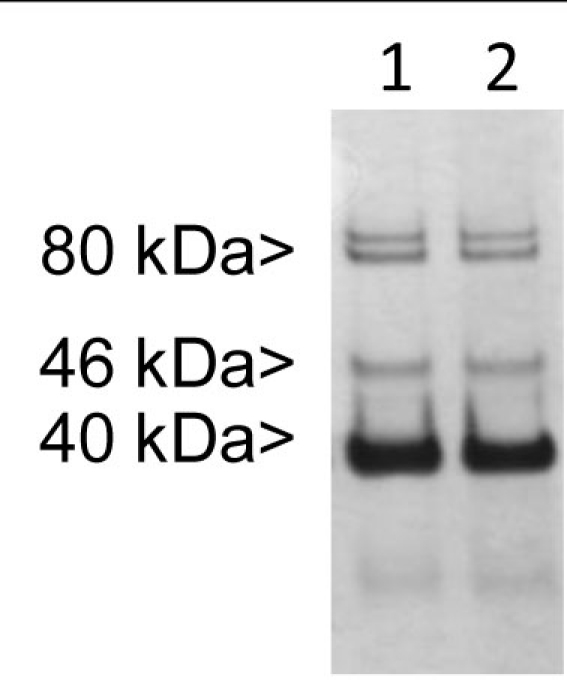
Immunoblotting analysis of 2M6 antigen purified from chick retina. Proteins obtained from embryonic day 13 chick retina were subjected to affinity purification using the 2M6-specific antibody conjugated to cyanogen bromide-conjugated (CNBr)-activated sepharose 4B. The eluate was subjected to SDS–PAGE and subsequent immunoblotting analysis using the 2M6-specific antibody. Signals were observed for polypeptides at ~40 kDa, ~46 kDa, and ~80 kDa. These polypeptides were subjected to in-gel trypsinization and subsequent mass spectrometry analyses. Lanes 1 and 2 show duplicate samples of the eluted fraction with the highest absorbance measured at 280 nm.

Affinity purification analyses were performed to determine the identity of the protein recognized by the 2M6 antibody. Previous reports have indicated that 2M6 is a glial-specific marker expressed within the developing chick retina [2-4]. Therefore, lysates from E13 chick retina were prepared for use. Immunoblotting analyses of the eluted fractions obtained revealed the presence of polypeptides of ~40 kDa, ~46 kDa, and a doublet of ~80 kDa (Figure 1). These polypeptides were subjected to in-gel trypsinization so that peptides could be recovered for MS/MS analyses.

**Figure 2:**
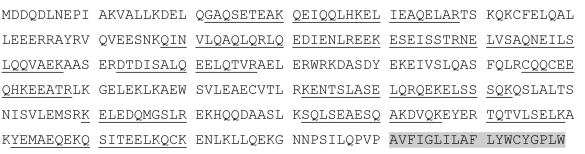
Amino acid sequence analysis of 2M6 antigen and Top_AP_ protein. Mass spectroscopy analyses identified 20 unique peptides, each of which align with the Top_AP_ polypeptide sequence (NCBI accession number gi|642486) with 95% probability. The Top_AP_ polypeptide sequence is shown, with the regions of identity to 2M6 peptides underlined. These regions account for 186 of the 359 amino acids, which is 52% coverage of the Top_AP_ sequence. The transmembrane region, located at the carboxy terminus of the polypeptide sequence, is highlighted in gray.

A total of 20 unique peptide sequences were obtained via mass spectrometry. A search of the NCBI protein database, using the peptide sequences as queries, revealed that the protein recognized by the 2M6 antibody is identical (95% probability) to a protein previously named Top_AP_. The peptide sequences obtained account for 186 of the 359 amino acids within the Top_AP_ molecule, which is 52% coverage. No other candidate proteins were identified as homologous to the sequenced peptides. The Top_AP_ (NCBI accession number gi|642486) sequence is shown with 2M6 peptides underlined (Figure 2). Hydropathy plot analyses of the sequence for 2M6 suggest that expression of the polypeptide is membrane associated [5]. The membrane-associated domain of the molecule, which is found at the carboxy terminus, is highlighted in Figure 2.

**Figure 3:**
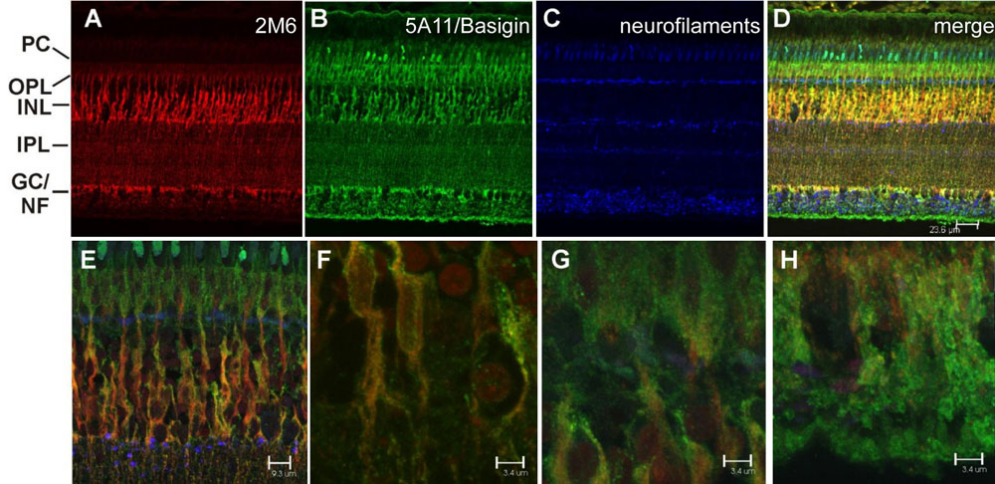
The 2M6 antigen is an intracellular protein expressed by Müller glial cells of the chick retina. Hatchling chick retina was fixed, embedded in paraffin, sectioned, and stained with antibodies specific for 2M6 (**A**, red) and 5A11/Basigin (**B**, green), and neurofilaments (**C**, blue). The overlay image (**D**) includes 4',6-diamidino-2-phenylindole (DAPI) staining of DNA (amber). Note that 2M6, which is expressed by Müller cells, is most heavily distributed between the ganglion cell layer and the outer limiting membrane. In contrast, 5A11/Basigin, also expressed by Müller cells, extends beyond the ganglion cells into the nerve fiber layer to the Müller cell endfeet. In panels **E** through **H**, higher magnification images compare and contrast the intracellular Müller cell membrane staining of 2M6 (red) with the Müller cell plasma membrane staining of 5A11/Basigin (green). In panel **E**, the Müller cell perikarial region of the inner plexiform layer is shown. In panel **F**, the Müller cell bodies are shown. Müller cell processes at the outer plexiform/photoreceptor cell layer are shown in panel **G**, whereas Müller cell processes at the ganglion cell/nerve fiber layer are shown in panel **H**. Abbreviations are as follows: PC represents the photoreceptor cell layer; OPL represents the outer plexiform layer; INL represents the inner nuclear layer; IPL represents the inner plexiform layer; GC/NF represents the ganglion cell/nerve fiber layer. The magnification bars for **A**–**D** are 23.6 μm; the magnification bar for **E** is 9.3 μm; the magnification bars for **F**–**H** are 3.4 μm.

**Figure 4:**
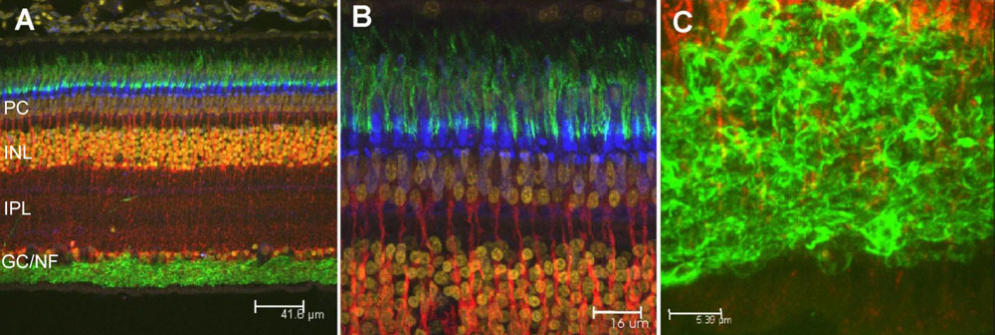
The 2M6 antigen is not expressed by neurons of the chick retina. Hatchling chick retina was fixed, embedded in paraffin, sectioned, and stained with antibodies specific for 2M6 (red) and calbindin (blue) and with peanut agglutinin (green). Panel **A** shows the full extent of the retina and retinal pigmented epithelium with 2M6 and peanut agglutinin signals merged with a 4',6-diamidino-2-phenylindole (DAPI; amber) labeling of cell nuclei. Panels **B** and **C** show higher magnification views at the outer and inner extremes of the neural retina, respectively. Abbreviations are as follows: PC represents the photoreceptor cell layer; INL represents the inner nuclear layer; IPL represents the inner plexiform layer; GC/NF represents the ganglion cell/nerve fiber layer. The magnification bar for **A** is 41.6 μm; the magnification bar for **B** is 16 μm; the magnification bar for **C** is 5.39 μm.

Since it has been reported that the 2M6 antigen is Muller-cell specific [3,4] and TopAP is thought to be a neuronal protein [5], immunohistochemical analyses were performed on sections of intact chick retina to better localize expression of this protein. An antibody specific for 5A11/Basigin was used to identify Müller cells of the chick retina [10,15]. An antibody specific for neurofilaments [11] as well as peanut agglutinin, which specifically stains cone outer sheathes [16], were used to identify neurons. Figure 3 demonstrates that 2M6 (red) expression overlaps that of 5A11/Basigin (green) and not neurofilaments (blue). Expression of 5A11/Basigin is observed throughout the entire Müller glial cells, whereas 2M6 expression is observed between the ganglion cell layer and the outer limiting membrane (Figure 3D). Higher magnification views of the expression patterns of 5A11/Basigin and 2M6 show that while 5A11/Basigin is found on the plasma membrane of Müller cells, 2M6 is found in the interior of the cell (Figure 3E–H). Figure 4 demonstrates that 2M6 (red) expression does not overlap that of peanut agglutinin (green) [16] or calbindin [12], which are neuronal markers in the retina. These findings clearly demonstrate that 2M6 (and hence Top_AP_) is a Müller cell-specific protein.

**Figure 5:**
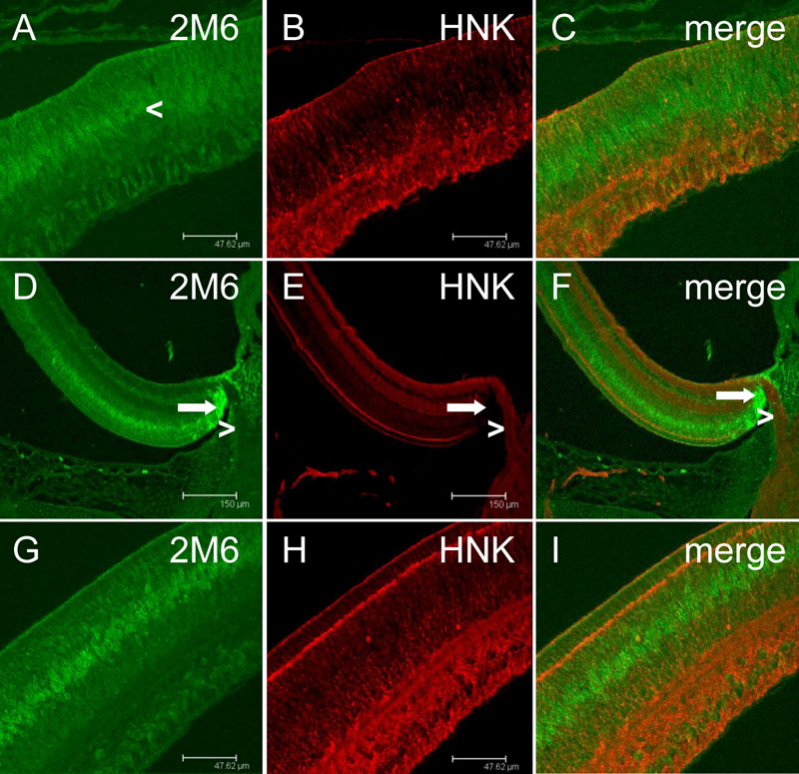
The 2M6 antigen has a graded expression pattern in the chick retina. Embryonic day 10 chick retina was fixed, embedded in paraffin, sectioned, and stained with antibodies specific for 2M6 (green) and human natural killer cell antigen (HNK; red). Three regions of the retina were examined, including the ciliary margin (**A**–**C**), optic nerve (**D**–**F**), and fundus (**G**–**I**), which represent the different stages of development within the retina at this age. Note that the orientation of panels **A**–**C** and **G**–**I** are dorso–ventrally flipped relative to that of **D**–**F**. At the ciliary margin, 2M6 expression changes from a diffuse pattern to one that is more specific (arrowhead, **A**). Boundary cells (arrows, **D**–**F**) are positively labeled with the 2M6-specific antibody, whereas optic nerve tracks (arrowhead, **D**–**F**) are labeled with the HNK cell-specific antibody. The magnification bars for **A**–**C** and **G**–**I** are 47.62 μm; the magnification bars for **D**–**F** are 150 μm.

Expression of 2M6 was also analyzed throughout the entire retina since Top_AP_ was reported to have a graded pattern of expression. E10 chick retinas were isolated, fixed, and embedded in paraffin for sectioning. Sections were chosen to show the different stages of differentiation within the chick retina at that age. The ciliary margin represents an early stage of retinal development with little cellular differentiation, whereas the fundus represents a late stage of development in which most cells have differentiated [17]. The region of the retina at the optic nerve, which represents an intermediate stage of development, was also examined. These three regions were probed with antibodies specific for 2M6 (red) and HNK (green). Diffuse-to-specific staining for 2M6 was observed at the ciliary margin, although HNK staining is specific throughout this region (Figure 5A–C). Boundary cells, a population of glia-like cells found at the junction of the neural retina and the optic nerve [18], robustly express 2M6 at this age (Figure 5D–F, arrows). In contrast, HNK is found within the ganglion cell processes leading from the neural retina (Figure 5D–F, arrowheads). Analysis of the fundus, which is the most differentiated region of the chick retina at this age [17], shows that 2M6 expression is not found in neurons as the signals for 2M6 and HNK do not overlap (Figure 5G–I). These data indicate that Müller cell-specific 2M6 expression does have a graded expression pattern in the developing chick retina.

**Figure 6:**
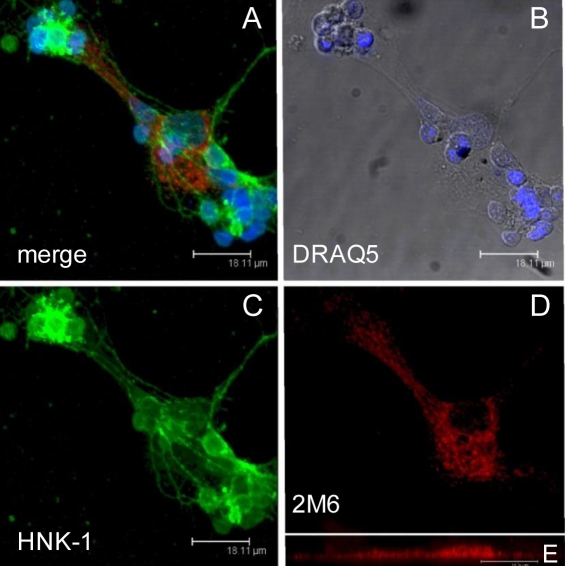
Analysis of dissociated chick retina indicates that 2M6 is a Müller cell-specific protein associated with internal membranes. Monolayer cultures from chick retina were prepared, fixed, and stained with antibodies specific for 2M6 (**D**, red) and human natural killer cell antigen (HNK; **C**, green), and with 1,5-bis{[2-(di-methylamino) ethyl]amino}-4, 8-dihydroxyanthracene-9,10-dione (DRAQ-5; **B**, blue). The overlay image (**A**) shows that 2M6-expressing and HNK-expressing cells are distinct from one another. No overlap of expression was observed, which indicates that 2M6 is not expressed by HNK-expressing neurons. Panel **E** is a computer-generated “side view” of the 2M6 signal showing that the reticulations within the Müller cell are distributed throughout the depth of the cell. A differential interference contrast image is also shown in panel **B**. The magnification bars for **A**–**D** are 18.11 μm; the magnification bar for **E** is 18.34 μm.

Immunohistochemical analyses were also performed on dissociated chick retina. Figure 6 shows a cluster of cells that are labeled with the antibody specific for 2M6 (red), an antibody specific for HNK, a neuronal cell-surface marker [13] (green), and DRAQ5, which highlights the cell nuclei (blue). An overlay of the three channels shows that 2M6 expression does not overlap that of HNK, which suggests that 2M6 is not a neuronal protein (Figure 6A). Also, when the Müller cell-derived flat cells adherent to the growth surface are viewed at high magnification, punctate 2M6 labeling is evidently associated with endomembranes of specific intracellular bodies (data not shown). The sequence data suggest that 2M6 is membrane associated. The immunohistological studies presented herein suggest that 2M6 is associated with internal membranes and not the plasma membrane of Müller cells. It has been demonstrated via electron microscopy that endoplasmic reticulum is widely distributed throughout Müller cells [19]. Figure 6E shows a computer-generated “side view” of a 2M6-expressing cell in which expression is observed throughout the depth of the cell. These data suggest that 2M6 is associated with Müller cell internal membranes, such as the endoplasmic reticulum.

## Discussion

Identification of cells in developing tissue is often accomplished through the use of the molecules expressed by those cells. The 2M6 antigen has been used as a differentiation marker for Müller glial cells of the vertebrate retina for many years [2-4]. Presently, we report that the amino acid sequence of the 2M6 antigen has been deduced by immunoaffinity isolation and subsequent mass spectrometry of peptides obtained by trypsinization. The peptides match with the amino acid sequence of Top_AP_, originally thought to be a neuronal marker. Immunohistochemical analyses of intact and dissociated chick retina indicate that 2M6 (Top_AP_) is indeed expressed by Müller cells.

The antibody specific for the 2M6 antigen has been used for some time as a Müller cell-specific marker [2-4], although the molecular identity of the antigen was not known. The purpose of this study was to isolate the 2M6 antigen via affinity purification for mass spectroscopy analyses. The peptides isolated were compared to known polypeptide sequences and all matched to the protein known as Top_AP_ (NCBI accession number gi|642486) with at least 95% probability. The Top_AP_ polypeptide is a member of the sarcolemmal membrane-associated protein (SLMAP) family of coiled-coil, tail-anchored, membrane proteins [20]. The SLMAP gene is rather large, consisting of 24 exons within 122 kb of DNA, located on human chromosome 3p14.3–21.2 [21-23]. There are several splice variants produced from the SLMAP gene, each possessing a central coiled-coil region containing two leucine zipper motifs and a C-terminal hydrophobic domain responsible for membrane localization of the polypeptides [21]. The Top_AP_ polypeptide consists of 359 amino acids [5] and is calculated to be ~40 kDa in molecular mass. The identities of the higher molecular mass species obtained via affinity purification (~46 and ~80 kDa) are not yet known, but their presence is consistent with the data reported by Savitt et al. [5] and by others working with members of the SLMAP and sarcolemmal-associated protein (an outdated name for this group) family [21]. It is therefore plausible to suggest that the higher molecular mass species obtained via affinity purification are splice variants of the 2M6 gene found in the retina. SLMAP splice variants have been found in the sarcolemma T-tubules and sarcoplasmic reticulum of cardiomyocytes as well as the microtubule-organizing centers (centrosomes) of mouse fibroblasts [22]. They have been implicated in organizing the excitation–contraction coupling apparatus and interacting with cardiac myosin in myocytes [23] as well as interacting with γ-tubulin within centrosomes [22]. Analysis of the 2M6 (Top_AP_) protein sequence using the SMART program [24,25] revealed the presence of a prefoldin domain, which is a leucine-zipper motif known to interact with α- and γ-tubulin [26,27], as well as a tropomyosin-like domain. Thus, the domain structures within the 2M6 (Top_AP_) polypeptide are consistent with functionalities within Müller cells that parallel those ascribed to SLMAP proteins in other cell types.

The immunohistochemical analyses presented herein indicate that 2M6 (Top_AP_) is indeed a glial-specific polypeptide. Both intact and dissociated chick retina preparations were examined for 2M6 expression and compared to the expression patterns of known neuron-specific markers. The expression of 2M6 does not localize with expression of neuronal markers, including neurofilaments [11], calbindin [12], peanut agglutinin [16], or HNK [13], in intact or dissociated chick retina. However, expression does correlate with the Müller cell-specific marker 5A11/Basigin [15]. Savitt et al. [5] did not clearly show that Top_AP_ was expressed by neurons of the developing chick retina but concluded this based on the graded expression pattern and recombinant antibody staining specific for Top_AP_ within the region of the optic nerve at a time when synapses form in the developing chick retina. There is a graded expression pattern, as suggested by Savitt et al. [5], in that the fundus, which is the most developed region of the retina [17], showed robust Müller cell-specific expression of 2M6, whereas fluorescence representing 2M6 expression was more diffuse at the ciliary margin (Figure 5). The data presented herein definitely indicate that neurons within the developing chick retina do not express 2M6 (Top_AP_) but that Müller cells do (Figure 3, Figure 4, and Figure 6). This Müller cell-specific expression of 2M6 is confined to the interior of the cell, as demonstrated in Figure 3, in which 2M6 expression is compared to that of plasma membrane-associated 5A11/Basigin. The 2M6 signal fills the interior of the cell and is reminiscent of electron micrographs depicting the distribution of endoplasmic reticulum throughout Müller cells [19].

Based on localization of expression and the conserved motifs found within the 2M6 polypeptide, it is tempting to speculate about the function of 2M6 in Müller cells. It has been documented that Ca^+2^ signaling occurs in Müller cells of the salamander retina such that Ca^+2^ is released from internal stores and flows from the apical end of the cell toward the endfoot in a wave-like motion [28]. These Ca^+2^ waves may provide a second signaling pathway from the outer to inner neural retina that is independent of neuronal signaling [29]. The 2M6 molecule may play a role in Ca^+2^ release from internal stores within Müller cells as the SLMAP isoform does in myocytes and perhaps interacts with both the cytoskeleton and the endoplasmic reticulum to accomplish this feat.

## References

[1] Livesey FJ Cepko CL Vertebrate neural cell-fate determination: lessons from the retina. Nat Rev Neurosci 2001 2 109 18 11252990 10.1038/35053522

[2] Schlosshauer B Grauer D Dütting D Vanselow J Expression of a novel Muller glia specific antigen during development and after optic nerve lesion. Development 1991 111 789 99 1879342 10.1242/dev.111.3.789

[3] Linser PJ Schlosshauer B Galileo DS Buzzi WR Lewis RC Late proliferation of retinal Müller cell progenitors facilitates preferential targeting with retroviral vectors in vitro. Dev Genet 1997 20 186 96 9216059 10.1002/(SICI)1520-6408(1997)20:3<186::AID-DVG2>3.0.CO;2-3

[4] Fischer AJ Scott MA Tuten W Mitogen-activated protein kinase-signaling stimulates Müller glia to proliferate in acutely damaged chicken retina. Glia 2009 57 166 81 18709648 10.1002/glia.20743 PMC2774719

[5] Savitt JM Trisler D Hilt DC Molecular cloning of TOPAP: a topographically graded protein in the developing chick visual system. Neuron 1995 14 253 61 7857637 10.1016/0896-6273(95)90283-x

[6] Institute of Laboratory Animal Research, Commission on Life Sciences, National Research Council. Guide for the Care and Use of Laboratory Animals. Washington, D.C.: The National Academies Press; 1996.

[7] Ochrietor JD Moroz TM Kadomatsu K Muramatsu T Linser PJ Retinal degeneration following failed photoreceptor maturation in 5A11/Basigin null mice. Exp Eye Res 2001 72 467 77 11273674 10.1006/exer.2000.0974

[8] Keller A Nesvizhskii AI Kolker E Aebersold R Empirical statistical model to estimate the accuracy of peptide identifications made by MS/MS and database search. Anal Chem 2002 74 5383 92 12403597 10.1021/ac025747h

[9] Nesvizhskii AI Keller A Kolker E Aebersold R A statistical model for identifying proteins by tandem mass spectrometry. Anal Chem 2003 75 4646 58 14632076 10.1021/ac0341261

[10] Linser PJ Perkins MS Regulatory aspects of the in vitro development of retina Müller glial cells. Cell Differ 1987 20 189 96 3568138 10.1016/0045-6039(87)90433-7

[11] Bennett GS Tapscott SJ DiLullo C Holtzer H Differential binding of antibodies against the neurofilament triplet proteins in different avian neurons. Brain Res 1984 304 291 302 6430468 10.1016/0006-8993(84)90333-0

[12] Chiquet C Dkhissi-Benyahya O Chounlamountri N Szel A Degrip WJ Cooper HM Characterization of calbindin-positive cones in primates. Neuroscience 2002 115 1323 33 12453500 10.1016/s0306-4522(02)00327-5

[13] Bakker H Friedman I Oka S Kawasaki T Nifant’ev N Schachner M Mantei N Expression and cloning of a cDNA encoding a sulfotransferase involved in the biosynthesis of the HNK-1 carbohydrate epitope. J Biol Chem 1997 272 29942 6 9368071 10.1074/jbc.272.47.29942

[14] Smith KE VanEkeris LA Linser PJ Cloning and characterization of AgCA9, a novel alpha-carbonic anhydrase from *Anopheles gambiae* larvae. J Exp Biol 2007 210 3919 30 17981859 10.1242/jeb.008342

[15] Fadool JM Linser PJ Differential glycosylation of the 5A11/HT7 antigen by neural retina and epithelial tissues in the chicken. J Neurochem 1993 60 1354 64 8455029 10.1111/j.1471-4159.1993.tb03296.x

[16] Blanks JC Johnson LV Selective lectin binding of the developing mouse retina. J Comp Neurol 1983 221 31 41 6643744 10.1002/cne.902210103

[17] Mey J Thanos S Development of the visual system of the chick I. Cell differentiation and histogenesis. Brain Res Brain Res Rev 2000 32 343 79 10760548 10.1016/s0165-0173(99)00022-3

[18] Linser PJ Irvin CK Immunohistochemical characterization of delta crystallin-containing retina/optic nerve “boundary” cells in the chick embryo. Dev Biol 1987 121 499 509 3108050 10.1016/0012-1606(87)90186-2

[19] Sarthy V, Ripps H, editors. The Retinal Muller cell: Structure and function. New York: Kluwer Academic/Plenum Publishers; 2001. p. 8.

[20] Wielowieyski PA Wigle JT Salih M Hum P Tuana BS Alternative splicing in intracellular loop connecting domains II and III of the alpha 1 subunit of Cav1.2 Ca^2+^ channels predicts two-domain polypeptides with unique C-terminal tails. J Biol Chem 2001 276 1398 406 11010971 11010971 10.1074/jbc.M006868200

[21] Wigle JT Demchyshyn L Pratt MA Staines WA Salih M Tuana BS Molecular cloning, expression, and chromosomal assignment of sarcolemmal-associated proteins. A family of acidic amphipathic alphα-helical proteins associated with the membrane. J Biol Chem 1997 272 32384 94 9405447 10.1074/jbc.272.51.32384

[22] Guzzo RM Sevinc S Salih M Tuana BS A novel isoform of sarcolemmal membrane-associated protein (SLMAP) is a component of the microtubule organizing centre. J Cell Sci 2004 117 2271 81 15126628 10.1242/jcs.01079

[23] Guzzo RM Salih M Moore ED Tuana BS Molecular properties of cardiac tail-anchored membrane protein SLMAP are consistent with structural role in arrangement of excitation-contraction coupling apparatus. Am J Physiol Heart Circ Physiol 2005 288 H1810 9 15591093 10.1152/ajpheart.01015.2004

[24] Schultz J Milpetz F Bork P Ponting CP SMART, a simple modular architecture research tool: identification of signaling domains. Proc Natl Acad Sci USA 1998 95 5857 64 9600884 10.1073/pnas.95.11.5857 PMC34487

[25] Letunic I Doerks T Bork P SMART 6: recent updates and new developments. Nucleic Acids Res 2006 34 D257 60 25300481 10.1093/nar/gku949 PMC4384020

[26] Shang HS Wong SM Tan HM Wu M YKE2, a yeast nuclear gene encoding a protein showing homology to mouse KE2 and containing a putative leucine-zipper motif. Gene 1994 151 197 201 7828874 10.1016/0378-1119(94)90656-4

[27] Geissler S Siegers K Schiebel E A novel protein complex promoting formation of functional alpha- and gamma-tubulin. EMBO J 1998 17 952 66 9463374 10.1093/emboj/17.4.952 PMC1170445

[28] Keirstead SA Miller RF Calcium waves in dissociated retinal glial (Müller) cells are evoked by release of calcium from intracellular stores. Glia 1995 14 14 22 7615342 10.1002/glia.440140104

[29] Newman E Reichenbach A The Müller cell: a functional element of the retina. Trends Neurosci 1996 19 307 12 8843598 10.1016/0166-2236(96)10040-0

